# Are Chimpanzees Really So Poor at Understanding Imperative Pointing? Some New Data and an Alternative View of Canine and Ape Social Cognition

**DOI:** 10.1371/journal.pone.0079338

**Published:** 2013-11-20

**Authors:** William D. Hopkins, Jamie Russell, Joe McIntyre, David A. Leavens

**Affiliations:** 1 Neuroscience Institute and Language Research Center, Georgia State University, Atlanta, Georgia, United States of America; 2 Division of Developmental and Cognitive Neuroscience, Yerkes National Primate Research Center, Atlanta, Georgia, United States of America; 3 Department of Psychology, University of Sussex, Sussex, England; CNR, Italy

## Abstract

There is considerable interest in comparative research on different species’ abilities to respond to human communicative cues such as gaze and pointing. It has been reported that some canines perform significantly better than monkeys and apes on tasks requiring the comprehension of either declarative or imperative pointing and these differences have been attributed to domestication in dogs. Here we tested a sample of chimpanzees on a task requiring comprehension of an imperative request and show that, though there are considerable individual differences, the performance by the apes rival those reported in pet dogs. We suggest that small differences in methodology can have a pronounced influence on performance on these types of tasks. We further suggest that basic differences in subject sampling, subject recruitment and rearing experiences have resulted in a skewed representation of canine abilities compared to those of monkeys and apes.

## Introduction

An important aspect of socio-communicative development in human children is the emergence of both the comprehension and production of pointing. Around 6 months of age, developing children begin to orient and follow human social communicative cues such as gaze and pointing [1], [2]. From around 12 to 15 months of age, the production of manual gestures emerges; these gestures are often directed to objects in the environment and accompanied by alternation of gaze between the referent and social agent [3], [4]. These early non-verbal communication abilities are an important stage in the development of a variety of cognitive and communicative abilities of human children. For instance, studies have shown that the age of onset of pointing is correlated with the onset of speech later in life [5], [6]. Furthermore, there is some evidence that delays in the onset or deficits in both the comprehension and production of pointing gestures are diagnostic of neurodevelopmental disorders such as specific language impairment and, notably autism spectrum disorder [7], [8].

From a comparative standpoint, studies in great apes and to a lesser extent monkeys have shown that they will use manual gestures to request food that is otherwise unattainable to them [9]–[11]. Further, ape gestures are sensitive to the presence of an audience, and are produced in conjunction with alternation of gaze between the referent and social agent, much like that has been described in developing human children [9], [12], [13]. From the comprehension perspective, studies in a variety of species have shown that they can follow gaze [14], [15] and to a lesser extent pointing [9], [16]–[18]. With regard to comprehension of pointing, there have been a number of reports suggesting that other species, notably dogs, perform significantly better than most primate species including great apes and monkeys [e.g., 19]. Specifically, initial studies reported that chimpanzees and other apes were poor at a specific pointing comprehension task, referred to as the object choice task (OCT). In the OCT, one of two or more opaque containers is baited with a food item. A human experimenter then points to the baited container, indicating which of the objects the subject should choose when provided with an opportunity to make a choice. In contrast to apes and monkeys, dogs perform quite well on the OCT and some have attributed these species differences to the influence of domestication or degree of socialization with humans [e.g., 19,20,21]. More recent studies have offered some more parsimonious explanations for the apparent differences between dogs and apes on the OCT, notably factors associated with the methodology and procedures used to assess OCT abilities between species [22], [23]. When comparable methods of OCT assessment are used, species differences in OCT performance dissipate.

In the current study, we address a more recent claim that dogs outperform chimpanzees on a version of the OCT in which the subjects are required to comprehend an imperative request [20], [24]. A distinction often made between the gestures of apes and human children is in their type and functional use; specifically, human children’s gestures are classified as (a) requestive (imperative) or (b) declarative in function. Imperative pointing is described as the instrumental use of gestures by individuals to request a specific action and object. In contrast, declarative pointing is defined as the motivation to indicate an event or object in the environment for the purposes of showing or, more richly, to share in joint attention. Within this framework, it has been hypothesized that human children produce both imperative and declarative pointing whereas apes and other animals only produce imperative pointing. This distinction has been previously used to account for the poor performance of chimpanzees on the OCT, the idea being that great apes do not understand the helpful intentions of experimenters who are pointing to baited containers [e.g., 20].

More recently, some have questioned not only the abilities of chimpanzees to comprehend declarative but also imperative gestures. Kirchhofer et al. [24] compared a sample of dogs and chimpanzees on a task not unlike the OCT but differed slightly in that the subjects were asked to retrieve one of two similar, but not identical objects that was requested by a human experimenter with, among other cues, a pointing gesture. In other words, the subjects were asked to return an object that was imperatively pointed to by a human experimenter. Kirchhofer et al. [24] reported that none of the 23 chimpanzees they tested were able to succeed on this task whereas 9 of the 32 dogs performed significantly better than chance. It should be noted that 73 dogs were recruited for this study, but only 32 (44%) displayed sufficient motivation to complete the tasks. In contrast, 20 of 23 chimpanzees (87%) completed testing. Thus, the dogs were significantly less likely to complete the experiment than were the chimpanzees (χ^2^(1, *N* = 96) = 13.10, *p*<.001), but the dogs were relatively more successful at retrieving the specific object requested, if they did complete testing. These authors interpreted these findings as evidence that domestication of dogs over time has resulted in their ability to detect the meaning and intent of human gestural commands.

The poor performance of the chimpanzees in the task used by Kirchhofer et al. [24] is somewhat surprising for two reasons. First, as noted above, there are numerous studies showing that chimpanzees and other great apes reliably produce imperative gestures [reviewed by 25,26]. It seems odd that chimpanzees would be capable of producing imperative gestures yet be incapable of comprehending them. Second, the poor performance by the chimpanzees is entirely inconsistent with a fairly large body of literature showing that apes can comprehend gestures, signs and even human speech cues [e.g., 27,28–32].

We have known for some time that apes can select specific objects from an array when presented with pointing gestures, acquired human signs and human spoken English words [e.g., 27,30,32]. For some examples, Gua, an infant chimpanzee, comprehended imperative points, accompanied by verbal commands, to close doors and to retrieve objects [30]. Furness [27] reported that a chimpanzee and an orangutan often followed his gaze to the correct target in an array, even when he did not want them to. Savage-Rumbaugh and colleagues [32] reported superior comprehension by Kanzi, a bonobo, of numerous spoken imperatives, when compared to a two-year-old child. Among the many spoken imperative sentences that Kanzi comprehended were “Put the telephone on the TV” and “Put the mushrooms in the cabinet.” There are numerous additional published examples of great apes correctly interpreting either imperative speech or imperative points, or both [e.g., 9,17,33–35] dating back to Witmer’s [36] observation, in clinically controlled conditions, that when Mrs. McArdle asked Peter, a chimpanzee, to “kiss papa”, he duly kissed Mr. McArdle. The proposal that a given species is capable of comprehending the referents of spoken, but not gestural, deictic imperatives is extraordinary, but the implications of this paradox were not developed by Kirchhofer et al. [24].

Given that others have shown that methodological factors play a critical role in the performance on the OCT in dogs compared to other animals, notably monkeys and apes [22], [23], in this study we examined whether similar methodological factors might influence the performance of chimpanzees on a task requiring that they understand a request gesture from a human experimenter. Rather than have the chimpanzees retrieve objects that were requested, we had our subjects respond to human pointing cues and return a single object to different specific locations, which were requested via human imperative pointing gestures. If chimpanzees are poor at comprehending human request gestures, as suggested by Kirchhofer et al. [24], then we hypothesized they would be equally poor on our task. Whereas their task involved selecting one of two objects and delivering it to an experimenter, our task involved selecting one object and delivering it to one of two or, later, three locations. Poor performance in this imperative task would support Kirchhofer et al.’s [24] interpretation that chimpanzees have difficulty understanding the referents of imperative points. Conversely, high performance by chimpanzees on the present version of the task would implicate sampling or other methodological factors in explaining the differences between their findings and ours.

## Methods

### Subjects

The subjects were 35 captive chimpanzees from the Yerkes National Primate Research Center (YNPRC) of Emory University. There were 25 female and10 males ranging in age from 15 to 44 years (*Mean* = 21.34 years, s.d. = 10.11). Though the subjects have been involved in a variety of behavioral and cognitive tasks over many years, none had been involved in the task used in this study. Indeed, several studies on communication in the YNPRC chimpanzees have described their ability to initiate joint attention [10], [13], [37]. All of the research conducted with the chimpanzees was approved by the Emory University Institutional Animal Care and Use Committee and followed the guidelines for ethical treatment of chimpanzees outlined by the Institute of Medicine. All the chimpanzees were housed in social groups ranging from two to 12 individuals. The chimpanzees are fed twice daily with a diet that consists of fruits, vegetables and commercially produced primate chow. Environmental enrichment, such as simulated tool use tasks or other non-nutritive substrates, were provided to the chimpanzees on a daily basis.

### Procedure

#### Pretraining

Most of the chimpanzees at the YNPRC will exchange objects for food items [e.g., 38] and we capitalized on this ability in the current study. Prior to testing, each chimpanzee received some training to assure that they understand the response demands of the task. At the onset of training, a single polyvinyl-choride (PVC) pipe (approx. 20 cm long and 4.5 cm in diameter) was placed on the outside mesh of the subject’s home cage. A small, round rock (approximately 2 cm in diameter) was then placed in the subject’s home cage, typically in front of the ape. The subject was then asked to return the rock by pointing with a cupped hand toward the rock. If the chimpanzee returned the rock, they received a secondary reinforcer (click of a clicker) followed by a small food reward (i.e. small piece of fruit or vegetable or a squirt of diluted juice). We next increased the response demand by teaching the subjects to place the rock inside the PVC pipe via successive approximation. The experimenter initially moved the tube in front of the rock as it was being returned. Then, the experimenter placed the PVC pipe in the mesh and left it stationary while pointing to the pipe and asking the subject to return the rock into the pipe. Once each subject successfully returned the rock through the opening in the stationary PVC pipe four to five times without errors, testing commenced.

#### Testing

**Figure 1:**
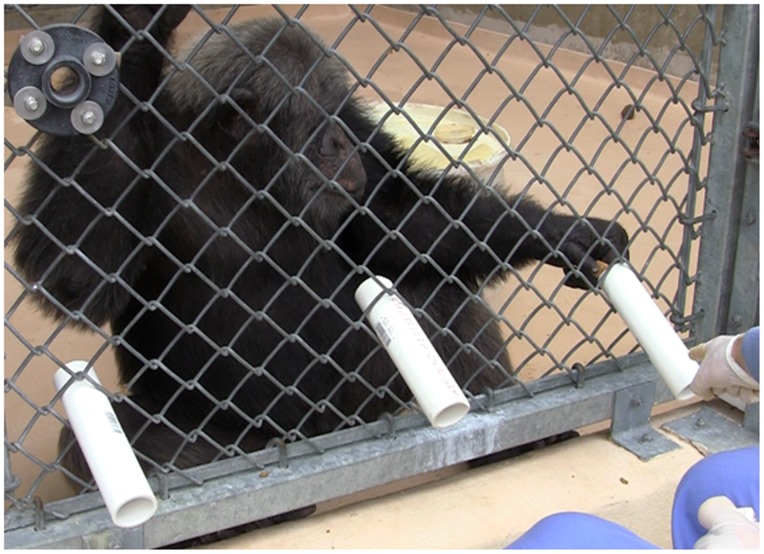
Photograph of the experimental test with PVC pipes placed in the cage and the human experimenter pointing to one of the pipes.

During the initial phase of testing, we placed two PVC pipes (approx. 20 cm long and 4.5 cm in diameter) in the cage mesh on the same horizontal plane, approximately 60 cm apart such that one end of the pipe was inside the enclosure and the rest of the pipe extended downwards out of the enclosure. A single rock (approximately 2 cm in diameter) was placed in the cage and the experimenter, sitting on a stool centered between the tubes, then pointed to one of the two PVC pipes, extending their arm and index finger such that the tip of their index finger was approximately 5–10 cm from the protruded end of the tube (approx. 25–30 cm from the end of the tube in which the chimpanzee placed the rock) while simultaneously saying the word “tube” (see Figure 1). The tube cued was pseudorandomized with the same tube never being repeated more than three consecutive trials. If the chimpanzee placed the rock in the correct PVC pipe (the one cued by the experimenter), they received a secondary reinforcer (click of a clicker) and a small food reward. Returning the rock to the wrong PVC pipe (the pipe not cued by the experimenter) resulted in no reward. After a short inter-trial-interval (3–5 seconds), the next trial commenced following the same procedure described above.

Each test session consisted of 20 trials with subjects receiving no more than 2 sessions per day (separated by a minimum of 2 hours) and subjects were tested repeatedly until they correctly placed the rock in the cued PVC pipe on 16 out of 20 trials, which was the established performance criterion. Upon completion of this initial testing, we further examined their generalization skills by adding a third PVC pipe during testing. The third pipe was placed approximately 35 cm below the horizontal plane of the first two tubes and centered between them vertically. The procedure was identical to the initial test phase with two PVC pipes except the experimenter would now cue to the chimpanzees to return the rock to one of the three possible PVC pipes located on the cage mesh. As in the two tube procedure, the selected tube was pseudorandomized with no tube being cued more than three consecutive trials. At the beginning of each three-tube testing session, the subjects received five warm-up trials with two tubes and were required to respond correctly in four or five of the five trials in order to proceed with testing. If they failed to respond correctly in at least 80% of the warm-up trials, 20 additional two-tube trials were completed. As in the two-tube tests, in the three-tube tests a correct response was recorded when the chimpanzee placed the rock in the cued PVC pipe. Correct responses were followed by a click and a food reward. Two dependent variables were of interest. First, we recorded the percentage of correct responses on the first test session in the two and three pipe conditions. Second, we recorded the number of test sessions needed to reach criterion for the two and three pipe conditions.

## Results

The individual data are shown in Table 1. For the two-PVC-pipe condition, a one-sample *t*-test showed that, overall, the chimpanzees performed significantly better than chance or 50% correct *t*(34) = 5.70, p<.001 (*Mean* = 66%). Of the 35 chimpanzees, the range in test sessions needed to reach criterion was 1 to 20 with just five subjects (5/35 or 14%) failing to reach criterion within 20 test sessions. Eleven chimpanzees performed significantly above chance (which required a performance of 75% correct or higher) on the very first test session. Neither sex nor rearing experience had a significant effect on Session 1 performance. For the three-PVC-pipe test, 30 chimpanzees were available for testing. As with the two-PVC-pipe condition, the chimpanzees performed significantly better than chance, which was 34%, on the first test session *t*(29) = 9.51, p<.001 (*Mean* = 71%). Twenty-four of the 30 chimpanzees (80%) responded significantly above chance (which required performance to be at 55% correct or higher) on the first test session when confronted with three PVC pipes. A comparison in the number of test sessions needed to reach criterion showed that the chimpanzee needed significantly fewer for the three- (*Mean* = 2.21) compared to two- (*Mean = *5.46) PVC-pipe test conditions *t*(29) = 3.13, *p*<.01. Thus, the chimpanzees showed improved performance on the task across testing conditions. As noted above, some chimpanzees received some “warm-up” or refresher trials before testing commenced on the three-PVC testing. To determine whether the warm-up testing had an impact on performance, we correlated the number of refresher tests needed for each chimpanzee with both session one performance and the number of test sessions needed to reach criterion in the three-PVC test. Seventeen chimpanzees passed the 5 trial warm up session on their first test while the remaining 13 needed between 1 and 6 additional warm up sessions. Not surprisingly, there was a significant negative association between the number of refresher sessions and session one performance (*r* = -.44, N = 30, *p*<.02.). Chimpanzees that needed fewer refresher trials did better on session one performance that those that needed more sessions. However, we found no significant association between the number of refresher trials needed and the number of trials to criterion for the three-tube test (*r* = .339, N = 30, n.s.).

**Table 1:** Individual Performance on the Two- and Three-PVC Test Conditions.

	Two PVC Pipes		Three PVC Pipes	
Subject	Session 1%	Tests	Session 1%	Tests
Abby	50	5	90*	1
Artemus	50	12	74*	2
Azalea	60	F		
Brandy	85*	1	75*	3
Brodie	50	4	90*	1
Callie	50	9	100*	1
Carl	60	3	95*	1
Cathy	50	20	25	4
Cissie	50	7	50	3
David	85*	1	55*	3
Elvira	75*	11	50	3
Faye	75*	2	95*	1
Fiona	85*	1	55*	1
Foxy	55	F		
Frannie	60	3	95*	1
Fritz	55	F		
Gelb	95*	1	55*	4
Jacqueline	70	15	85*	1
Julie	65	5	65*	4
Katrina	100*	1	100*	1
Lamar	70	2	100*	1
Lil’One	50	F		
Liza	50	19	80*	1
Lucas	50	4	100*	1
Melissa	100*	1	80*	1
Patrick	75*	2	60*	3
Rebecca	100*	1	80*	1
Rita	65	6	55*	2
Sabrina	50	5	40	5
Scott	85*	1	85*	1
Shirley	65	7	45	4
Socrates	55	20	55*	5
Sylvia	60	4	45	2
Tara	60	5	60*	3

F = failed to reach criterion within 20 test sessions.

* indicates significantly better than chance performance during session 1 tests.

## Discussion

The result reported here are straightforward. Though there were considerable individual differences within our sample, the chimpanzees clearly demonstrated significant competencies in their comprehension of the referents of imperative gestures. Nearly one-third of the chimpanzees could perform the task with no specific training and nearly 85% of the subjects learned to perform the task within the 20 test-session criterion. Furthermore, most chimpanzees showed generalization in performance in the task when the number of response options increased from two to three. Thus, our apes far exceeded the performance of the chimpanzees and, indeed, rivaled the results reported in dogs by Kirchhofer et al. [24]. These results directly challenge the claim that chimpanzees do not understand the referents of imperative pointing and raise additional questions regarding purported mechanisms underlying species differences in performance on object-choice or related types of tasks.

We are not surprised by the findings reported here and believe that some of the arguments and methodological assumptions regarding the abilities of dogs and chimpanzees on the OCT and its variants warrant critical analysis. Specifically, as noted by others [39] and reinforced here, the methods and approaches used to evaluate OCT performance can have a significant effect on performance in different species. A simple change in the manner that OCT performance was assessed in this study, compared to that used by Kirchhofer et al. [24], had a significant impact on the chimpanzees’ performances. In short, methodological factors play a greater role in explaining individual differences in OCT performance than either species or other purported mechanisms such as domestication.

We do not believe that there is anything special about our chimpanzees that accounts for their performance but, rather, we simply designed the task around abilities that have been well documented in chimpanzees. There are a host of previous studies that have shown that chimpanzees will exchange objects or tokens for food offered by humans [e.g., 38,40]. It seems logical that in order for chimpanzees to learn to perform such behaviors they must understand something about request gestures. It might be suggested that the pretraining the chimpanzees received on placing the rock in a single tube, prior to the subsequent initial tests may have influenced their performance but we do not believe this to be the case. Unfortunately, we did not record the number of pretraining sessions the chimpanzees received prior to the onset of the two-PVC-pipe condition. Therefore we cannot fully rule out this possibility but we would subjectively note that very few chimpanzees needed much pretraining given their previous experiences in bartering with humans. Additionally, the assumption is that the pretraining had a facilitative effect on subsequent performance on the two-PVC-pipe conditions but, arguably, the case could be made that it could have an inhibitory effect on performance. In this case, pretraining on the placement of a single rock into the PVC pipe would have reinforced the canalization of this response without consideration of the need to attend to social cues in order to place the rock in the correct tube in the two-PVC-pipe condition.

As noted above, the very claim that chimpanzees do not comprehend human pointing and requesting has been refuted for quite some time, going all the way back to some of the early ape language studies [e.g., 27,30,36]. None of this research was cited in the paper by Kirchhofer et al. [24], despite the fact that they challenge the very foundation and rationale for their study. Of course, the argument might be made that the so-called language-trained chimpanzees are not a fair or legitimate comparison group for discussion of basic OCT skills in chimpanzees because they have had extensive interactions with humans and may have been inadvertently “trained” to perform such tasks. As far as we know, however, humans require the same extensive interactions with other humans before they display the same sorts of competencies, therefore this argument is invalid [e.g., 26]. For example, typically developing humans do not develop this comprehension until well into their second year of life [e.g., 1]. If it could be demonstrated that human children develop the ability to comprehend both spoken and gestural referents despite being isolated from typical human interactions, then this argument might hold, but we are unaware of any such demonstration. To the contrary, children raised in austere institutional settings display global sociocognitive deficiencies [e.g., 41]. Moreover, Kirchhofer and her colleagues [24] made no apparent attempt to isolate the dogs in their study from extensive interactions with humans, therefore, the groups were not matched on this critical life history variable. This observation highlights a significant problem with current attempts to compare the cognitive abilities of pet dogs with zoo- and laboratory-living apes. Specifically, dogs have been selectively bred for the purposes of co-existence with humans but more importantly, most dogs in cognitive studies are pets and have extensive backgrounds with their owners. From this perspective, the only chimpanzees that should be compared to pet dogs are those that have extensive experience with humans. Zoo- or sanctuary-living chimpanzees, even those with extensive research backgrounds, are not a valid comparison group to a domesticated sample of pet dogs.

The problem with using pet dogs for comparison to chimpanzees (or nearly any other species) is not limited to the degree of experience or domestication they have with their human companions. More problematic is the simple fact that pet dogs used in cognitive research are not randomly selected. There are at least two particularly salient sampling problems that afflict this line of research. First, researchers studying pet dogs, including the studies by Kirchhofer et al. [24], recruit dog subjects by advertising and the owners then sign their pets up to serve as subjects in this research. This raises the question: who signs up their dog to be in a research project on canine cognition? In all likelihood, these are owners who have dogs that typically are very human oriented and are likely to do well on these kinds of tasks. This, by itself, would not be problematic, but the chimpanzees (or again any other species) are randomly selected. Chimpanzees or other species used for comparison to pet dogs are selected by convenience. In the present study, or in the study by Kirchhofer et al. [24], the chimpanzee subjects were selected because they reside at YNPRC or other research facilities and were available for use. In our view, this significant difference in subject selection makes comparisons between pet dogs and conveniently sampled chimpanzees illegitimate.

The second obvious contemporary sampling bias with pet dogs, possibly a consequence of the first, is that dog breeds are not randomly sampled within the species. Thus, of the 32 dogs in their study, fully 27 (84%) were from working dog breeds, including retrievers or retriever mixes (*n* = 14) and an additional 13 (40%) other working dog breeds, such as border collies, German shepherds, German pointers, and so on. Only 5 dogs (16% of their sample) were from non-retrieving, non-working breeds. Thus, although it is clear from their study that retrievers are very good at retrieving, the dog sample in Kirchhofer et al. [24] was very far from being representative of the range of extant dog breeds, and therefore their results do not even generalize to all dogs. The problem of selection bias for pet dogs has recently been illustrated in several studies that examined OCT performance in pet dogs compared to a “random” sample of dogs living in animal shelters [21], [42]. In these studies, the shelter dogs perform more poorly than the pet dogs and this has been attributed to variation in their experience with humans. We would suggest that it is also just as plausible that shelter dogs are a more representative sample of canine capacities for the OCT performance and that pet dogs represent a highly biased group of individuals and do not reflect the inherent abilities of dogs for the OCT.
